# Molecular and physiological characterization of the effects of auxin-enriched rootstock on grafting

**DOI:** 10.1038/s41438-021-00509-y

**Published:** 2021-04-01

**Authors:** Longmei Zhai, Xiaomin Wang, Dan Tang, Qi Qi, Huseyin Yer, Xiangning Jiang, Zhenhai Han, Richard McAvoy, Wei Li, Yi Li

**Affiliations:** 1 Department of Plant Science and Landscape Architecture, University of Connecticut, Storrs, CT 06269 USA; 2 College of Horticulture, China Agricultural University, Beijing, 100193 PR China; 3 Institute of Botany, Jiangsu Province and Chinese Academy of Sciences, Nanjing, 210014 PR China; 4 National Engineering Laboratory for Tree Breeding, College of Life Sciences and Biotechnology, Beijing Forestry University, Beijing, 100083 PR China

**Keywords:** Molecular engineering in plants, Auxin

## Abstract

Grafting is a highly useful technique, and its success largely depends on graft union formation. In this study, we found that root-specific expression of the auxin biosynthetic gene *iaaM* in tobacco, when used as rootstock, resulted in more rapid callus formation and faster graft healing. However, overexpression of the auxin-inactivating *iaaL* gene in rootstocks delayed graft healing. We observed increased endogenous auxin levels and auxin-responsive *DR5::GUS* expression in scions of WT/*iaaM* grafts compared with those found in WT/WT grafts, which suggested that auxin is transported upward from rootstock to scion tissues. A transcriptome analysis showed that auxin enhanced graft union formation through increases in the expression of genes involved in graft healing in both rootstock and scion tissues. We also observed that the ethylene biosynthetic gene *ACS1* and the ethylene-responsive gene *ERF5* were upregulated in both scions and rootstocks of the WT/*iaaM* grafts. Furthermore, exogenous applications of the ethylene precursor ACC to the junction of WT/WT grafts promoted graft union formation, whereas application of the ethylene biosynthesis inhibitor AVG delayed graft healing in WT/WT grafts, and the observed delay was less pronounced in the WT/*iaaM* grafts. These results demonstrated that elevated auxin levels in the *iaaM* rootstock in combination with the increased auxin levels in scions caused by upward transport/diffusion enhanced graft union formation and that ethylene was partially responsible for the effects of auxin on grafting. Our findings showed that grafting success can be enhanced by increasing the auxin levels in rootstocks using transgenic or gene-editing techniques.

## Introduction

Grafting is an ancient but essential technique used worldwide in the agricultural industry that involves combining plants of the same or different species to continue their growth and development^1^. In the horticultural and silvicultural industries, grafting is widely used to increase the yield, enhance biotic and abiotic stress resistance, and modify the scion architecture^2,3^. Although grafting has been used for millennia in agriculture, the mechanisms underlying the graft healing process are largely unclear. Previous studies have described the initial cell proliferation that occurs at the beginning of graft union formation, and this step is followed by the formation of a mass of pluripotent, undifferentiated cells known as calli. The callus then differentiates into vascular tissues to reconnect the phloem and xylem across the graft junction, which leads to a successful graft union^4,5^.

Various plant hormones are involved in the graft healing process. Auxin accumulates above the graft junction and is depleted at the bottom of the junction^5^. Exogenous auxin application can lead to successful graft formation during tissue culture^6^. Conversely, hypocotyl graft unions are inhibited in response to treatment with an auxin transport inhibitor^7^. Indeed, many *Arabidopsis thaliana* mutants with impaired auxin perception or auxin responses show delayed phloem reconnection during hypocotyl grafting^4^.

Transcriptome analyses have uncovered a series of auxin response genes involved in the grafting process^8,9^. The auxin response factors ARF6 and ARF8 are essential for cell proliferation during tissue reunion. The *arf6 arf8* double mutant shows strongly inhibited cellular proliferation during tissue reunion in incised *Arabidopsis* inflorescence stems compared with that obtained with the wild type^10^. Another important gene for grafting, *ABERRANT LATERAL ROOT FORMATION 4 (ALF4)*, acts downstream of auxin signaling and is specifically required in the rootstock for normal phloem reconnection during graft union formation^4^. Moreover, auxin induces the expression of the xyloglucan endo-transglucosylase/hydrolase genes *XTH20* and *XTH19*, which encode proteins involved in cell proliferation during the tissue reunion process, in the distal parts of incised stems^11,12^.

Auxin also interacts with other hormones during graft union formation^5^. Ethylene is involved in the wounding response and promotes cell expansion^13,14^, and the exogenous application of ethylene promotes callus formation to result in increased cell proliferation^15^. The ethylene signaling-defective *Arabidopsis* mutant *ein2* shows incomplete healing during tissue reunion, and incised inflorescence stems of this mutant exhibit inhibited expression of the transcription factor gene *ANAC071*, which is needed for the division of pith cells during reunion. The expression of *ANAC071* is also induced by auxin during this process^11^. Moreover, the application of the ethylene biosynthesis inhibitor AVG (2-aminoethoxyvinylglycine) to auxin (indole-3-butyric acid [IBA] or naphthalene acetic acid [NAA])-supplemented medium inhibits callus formation at the base of the shoot^15^. These findings indicate that ethylene and auxin interact during graft union formation, but further studies are needed to elucidate the detailed mechanism.

Here, we used auxin-overproducing transgenic tobacco, in which *iaaM* (one tryptophan-2-monooxygenase gene from *Agrobacterium tumefaciens*) is expressed predominantly in roots, as the rootstock for grafting to a wild-type (WT) scion to study the role of endogenous auxin in the graft healing process. This *Agrobacterium* gene *iaaM*, when inserted into plants, can convert tryptophan to indole-3-acetamide, and this product is then converted to the active phytohormone indole-3-acetic acid (IAA) by endogenous hydrolases^16^. The overexpression of *iaaM* in rootstock led to an increase in the auxin levels and upregulated the expression of genes associated with graft union formation in WT scions. Our findings indicate that ethylene promotes graft union formation and is involved in rapid auxin-mediated callus formation during the graft healing process.

## Results

### Altered auxin levels affect graft healing

**Fig. 1:**
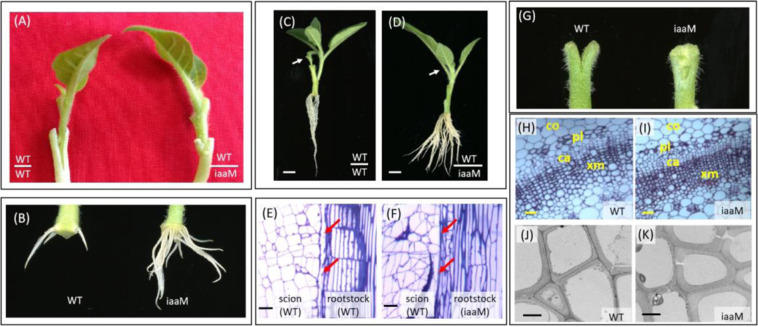
The use of *iaaM* rootstock improves graft healing. **A** One week after grafting, complete graft healing was observed in the WT/*iaaM* grafts, whereas only partially healed graft unions with obvious deeper gaps developed in the WT/WT grafts. **B** One week after grafting, the *iaaM* rootstock exhibited earlier root initiation and more and longer roots than the WT rootstock. **C**, **D** Two weeks after grafting, the WT/*iaaM* grafts (**D**) showed enhanced root biomass, more vigorous scion growth, and no lateral bud release compared with the WT/WT grafts (**C**). The arrows point to scions; Bar = 1 cm. **E**, **F** A microscopic analysis performed two weeks after grafting showed complete healing in the WT/*iaaM* grafts (**F**), whereas obvious gaps were found between the scion and rootstock in the WT/WT grafts (**E**). The arrows point to the graft junctions; Bar = 200 μm. (**G**) One week after longitudinal cutting, the WT stems produced little callus, and the split remained unhealed; in contrast, the cut *iaaM* stems completely healed, and a mass of callus cells filled the gap. **H**, **I** The *iaaM* stems contained more cambium and xylem cells (**I**) than the WT stems (**H**); Bar = 300 μm. **J**, **K** The xylem cells of the *iaaM* stems had thicker cell walls (**K**) than those of the WT stems (**J**); Bar = 100 μm. ca, cambium; co, cortex; pl, phloem; xm, xylem

The *iaaM* gene driven by the root-predominant promoter *SbUGT*, which is a flavonoid glycosyltransferase gene from *Scutellaria barbata*^17^, is expressed predominantly in the roots of tobacco (Fig. S1A, B). In this study, we used *SbUGT*::*iaaM* (*iaaM*) plants as the rootstock for grafting studies. One week after grafting, we observed complete graft healing with no gaps in the WT/*iaaM* grafts but only partially healed graft unions with clearly deeper gaps in the WT/WT grafts (Fig. 1A). In addition, the *iaaM* rootstocks exhibited earlier root initiation and more and longer roots than the WT rootstocks (Fig. 1B). Two weeks after grafting, the WT/*iaaM* grafts showed enhanced root biomass, more vigorous scion growth, and no lateral bud release compared with the WT/WT grafts (Fig. 1C–D). Three weeks after grafting, we analyzed the graft success rates of the two graft combinations. The graft success rate of the WT/*iaaM* grafts was 96.7%, whereas a success rate of only 75.9% was obtained for the WT/WT grafts (Table 1). We conducted a microscopic analysis of the graft unions of both the WT/WT and WT/ *iaaM* grafts, and the results showed complete healing in the WT/*iaaM* grafts, whereas obvious gaps were observed between the scion and rootstock in the WT/WT grafts (Fig. 1E, F).

**Table 1:** Grafting success rates

Graft (scion/rootstock)	Grafting success rate (%)
**(Mean** ± **SE)**	
WT/WT	75.9 ± 4.9
WT/*iaaM*	96.7 ± 3.0*

Note: Grafts with more than 1-cm increases in scion growth were considered successfully grafted. The data were collected three weeks after grafting. The experiment was repeated three times. Each replicate included five to eight grafts. The asterisks (*) indicate a significant difference between the WT/WT and WT/*iaaM* grafts, as determined using two-tailed Student’s t-test with pooled variance.

We further prepared stem sections with a 0.5-cm longitudinal cut from the top of each section using WT and *iaaM* tissue to investigate the *iaaM*-mediated improvement of graft healing in more details. One week after longitudinal cutting, the WT stems produced little callus, and the split remained unhealed; in contrast, the cut *iaaM* stems were completely healed, and a mass of callus cells filled the gap (Fig. 1G). Callus formation is a basic wound response to grafting^18^. Reduced callus production at the WT/WT graft junction might lead to graft failure, whereas our results suggest that rapid callus formation in the *iaaM* rootstock might improve graft union formation. We prepared transverse sections of the WT and *iaaM* stems to study the cellular characteristics of the vascular system. The *iaaM* stems showed more cambium and xylem cells than the WT stems (Fig. 1H, I), and the xylem cells of the *iaaM* stems had thicker cell walls than those of the WT stems (Fig. 1J, K); these properties might help strengthen the adhesion between the scion and rootstock and thereby facilitate graft healing.

**Fig. 2:**
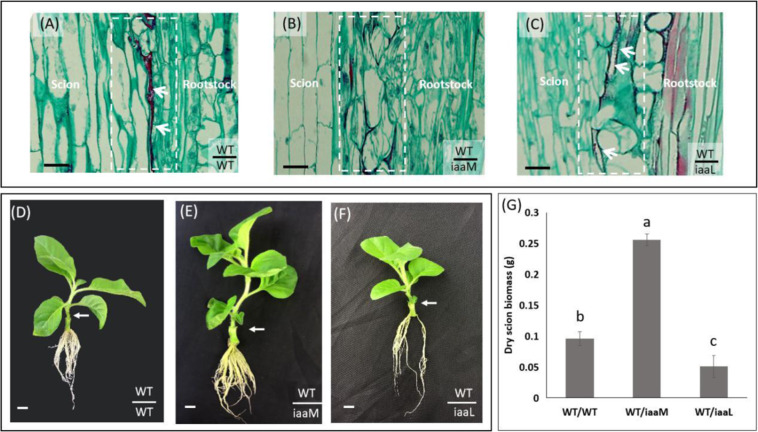
The use of *SAUR::iaaL* (an auxin-inactivating gene strongly expressed in stem tissues) rootstocks delays graft healing. **A**–**C** One week after grafting, larger gaps were observed in the unions of the WT/*iaaL* grafts (**C**) than in those of the WT/WT grafts (**A**). Among the grafting combinations (WT/WT, WT/*iaaM*, and WT/*iaaL*), the WT/*iaaM* grafts exhibited the best healing (**B**). The white arrows point to unhealed gaps between scions and rootstocks, and the dotted white rectangles indicate graft junction regions. Bar = 200 μm. **D**–**F** Three weeks after grafting, scion growth was faster in the WT/*iaaM* grafts (**E**) than in the WT/WT grafts (**D**), but slower in the WT/*iaaL* grafts (**F**) than in the WT/WT grafts (**D**). The white arrows point to the junctions of each graft combination. Bar = 1 cm. **G** Compared with the WT/WT grafts, the WT/*iaaM* grafts showed significantly higher scion growth, whereas the WT/*iaaL* grafts presented significantly reduced scion biomass. The data are presented as the means from three independent biological replicates. Bars with different letters are significantly different at *P* < 0.05 (ANOVA, LSD). The bars show standard deviations

To explore the role of endogenous auxin in the rootstock in the grafting process, we used transgenic plants harboring the auxin-inactivating indoleacetic acid-lysine synthetase (*iaaL*) gene driven by the shoot-specific *SAUR* promoter^19^. The *iaaL* gene encodes an indole-3-acetic acid-lysine synthetase that catalyzes the conversion of free auxin into inactive indole-3-acetyl-L-lysine^20^. Here, we used *SAUR::iaaL* (*iaaL*) plants as the rootstock for WT scions. The reduced endogenous auxin levels caused by *iaaL* expression in rootstocks exerted severe effects on graft healing. One week after grafting, we observed larger gaps in the unions of the WT/*iaaL* grafts than in those of the WT/WT grafts. Among the graft combinations (WT/WT, WT/*iaaM*, and WT/*iaaL*), the WT/*iaaM* grafts exhibited the best healing (Fig. 2A–C). Three weeks after grafting, scion growth was faster in the WT/*iaaM* grafts than in the WT/WT grafts but slower in the WT/*iaaL* grafts than in the WT/WT grafts (Fig. 2D–F). We also measured the scion biomass of all three grafts. Compared with the WT/WT grafts, the WT/*iaaM* grafts showed significantly higher scion biomass, whereas the WT/*iaaL* grafts presented significantly reduced scion biomass (Fig. 2G). Therefore, the use of *iaaL* rootstock caused a delay in graft healing and reduced scion growth, which further confirmed the role of endogenous auxin in the graft healing process.

### Auxin transported upward from rootstocks to scions enhances graft healing-related gene expression in scions

**Fig. 3:**
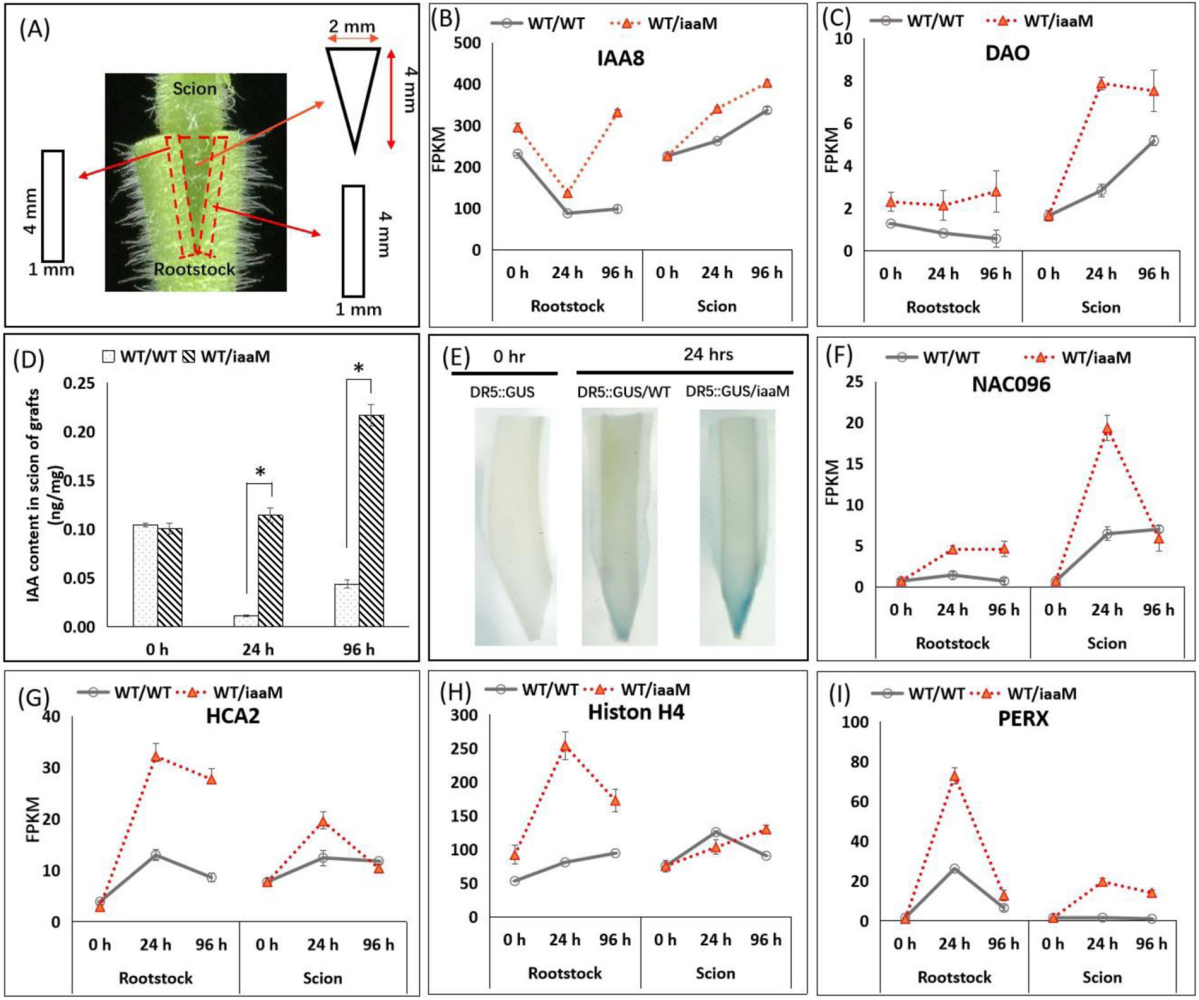
The use of *iaaM* rootstocks results in higher auxin accumulation in scions, as indicated by increased expression of auxin-responsive genes and increased auxin concentrations. **A** Scion and rootstock tissues were separately collected from the WT/WT and WT/*iaaM* grafts for RNAseq analysis. The rectangles represent tissues collected from rootstocks, and the inverted triangles represent tissues collected from scions. **B**, **C** The expression levels of the auxin-responsive genes *IAA8* (**B**) and *DAO* (**C**) were higher in both the scion and rootstock of the WT/*iaaM* grafts than in those of the WT/WT grafts at 24 h and 96 h after grafting. The gene expression levels were calculated using fragments per kilobase of transcript sequence per million base pairs sequenced (FPKM). The data are presented as the means from three independent biological replicates, and representative replicates were confirmed by qPCR analysis. The bars show standard deviations. **D** The IAA contents were significantly higher in the WT/*iaaM* scions than in the WT/WT scions at both 24 and 96 h after grafting. The data are presented as the means from three independent biological replicates. The bars show standard deviations. The asterisks (*) indicate a significant difference, as determined using two-tailed Student’s t-test with pooled variance. **E** The *DR5::GUS* scions exhibited more GUS activity on the *iaaM* rootstocks than in the WT rootstocks. “0 h” refers to scion and rootstock tissues collected immediately after cutting. **F** *NAC096*, an auxin-inducible gene that promotes cell division; **G** *HCA2*, a vascular development-related gene; **H** *Histone H4*, a cell division-promoting gene; and **I** *PERX*, a gene that facilitates cell wall thickening. “0 h” refers to scion and rootstock tissues collected immediately after cutting. The gene expression levels were calculated as FPKM values. The data are presented as the means from three independent biological replicates, and representative replicates were confirmed by qPCR analysis. The bars show standard deviations

We conducted a transcriptomic analysis to explore the molecular mechanisms underlying the rapid callus formation and fast graft healing observed with the use of *iaaM* plants as the rootstock. We harvested scion and rootstock samples from both WT/WT and WT/*iaaM* grafts at 0, 24, and 96 h after grafting (Fig. 3A). In the rootstock of the WT/*iaaM* grafts, the expression levels of auxin-responsive genes, such as *IAA8* and *DAO* (*DIOXYGENASE of AUXIN OXIDATION*), were higher at both 24 and 96 h after grafting than in the rootstock of the WT/WT grafts (Fig. 3B, C), which suggested that the auxin levels in the WT/*iaaM* rootstock were higher. Moreover, in the scions of WT/*iaaM* grafts, we detected markedly increased expression of these two auxin-responsive genes, which suggested that the increased endogenous auxin levels in rootstock might also lead to increased auxin levels in scion tissues. To demonstrate the biological significance of the increased endogenous auxin levels in the scion tissues of *iaaM* rootstock, we analyzed the free IAA content in the scions of the WT/WT and WT/*iaaM* grafts. The IAA concentrations in the WT/*iaaM* scions were significantly higher than those in the WT/WT scions at both 24 and 96 h after grafting, as demonstrated by increases of 10.6-fold and 5.0-fold, respectively (Fig. 3D). We also grafted the auxin-responsive element *DR5*::*GUS* transgenic tobacco onto *iaaM* and WT plants. Twenty-four hours after grafting, the basal ends of the *DR5*::*GUS* scions exhibited higher GUS activity on the *iaaM* rootstock than in the WT rootstock (Fig. 3E, Fig. S2), which indicated that the *iaaM* rootstock induced higher levels of auxin accumulation in scions than the WT rootstock. These findings suggest that the increased auxin levels in the *iaaM* rootstock led to high levels of auxin accumulation in scions and thereby facilitated graft union formation.

Furthermore, the expression levels of a series of genes related to graft healing, vascular system formation, and cell wall modification were increased 24 h after grafting in the rootstocks of both the WT/*iaaM* and WT/WT grafts, although a more pronounced increase was observed in the *iaaM* rootstock (see Table S1). For instance, *NAC096*, which encodes a NAC domain-containing protein needed for the auxin-mediated promotion of vascular tissue proliferation^7^, was expressed at 3.2-fold higher levels in the *iaaM* rootstock than in the WT rootstock (Fig. 3F). In addition, *HCA2* (*HIGH CAMBIAL ACTIVITY2*), which is critical for phloem reconnection^8^, showed 2.5-fold higher expression in the *iaaM* rootstock than in the WT rootstock (Fig. 3G). Additionally, the expression levels of both the cell division-promoting gene *Histone H4* and the peroxidase-encoding gene *PERX*, which facilitates cell wall thickening, were significantly higher in the *iaaM* rootstock than in the WT rootstock (Fig. 3H, I). These results are consistent with our observation that the *iaaM* rootstock contained more cambium cells and thicker xylem cell walls than the WT rootstock. The expression levels of these genes were also higher in scions of the *iaaM* rootstock than in those of the WT rootstock (Fig. 3F–I), which suggested that auxin enhances graft union formation by increasing the expression of genes involved in graft union formation in both rootstock and scion tissues.

### Ethylene is involved in the action of auxin during grafting

**Fig. 4:**
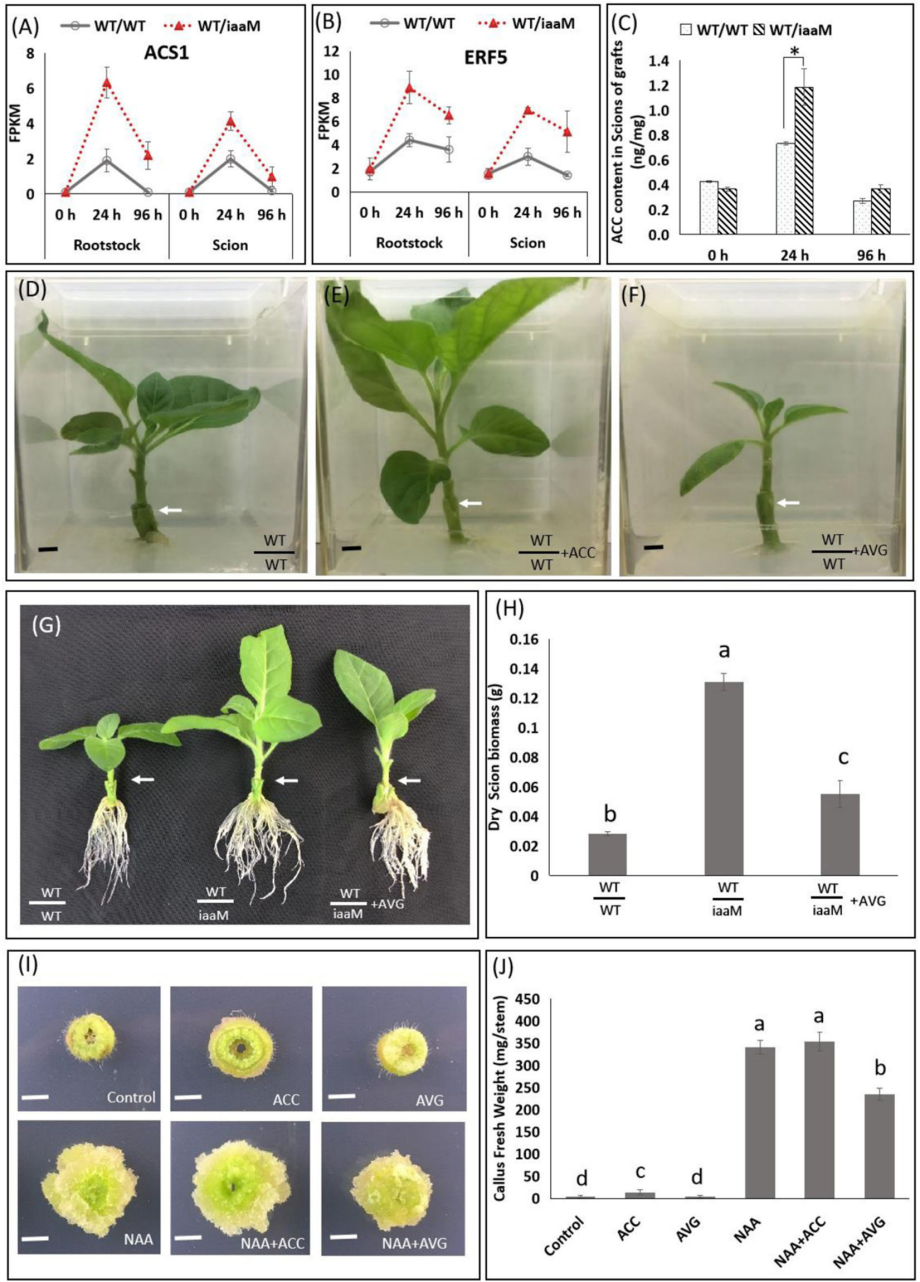
Ethylene is a positive regulator of grafting. **A**, **B** The ethylene biosynthesis-related gene *ACS1* (**A**) and the ethylene-responsive gene *ERF5* (**B**) were upregulated in the junction cells of the scions and rootstocks of the WT/*iaaM* grafts 1 day after grafting. “0 h” refers to scion and rootstock tissues collected immediately after cutting. The gene expression levels were calculated as FPKM values, and representative gene expression levels were confirmed by qPCR analysis. **C** The ACC levels in the WT/*iaaM* scions were significantly higher than those in the WT/WT scions 24 h after grafting. **D**–**F** Three weeks after grafting, scion growth was enhanced by the exogenous application of the ethylene precursor ACC (**E**) but reduced by the applications of the ethylene inhibitor AVG (**F**) compared with the levels found with the control treatment (**D**). **G**, **H** Three weeks after grafting, the ethylene biosynthesis inhibitor AVG reduced scion growth in the WT/*iaaM* grafts. **I**, **J** Three weeks after treatment with the synthetic auxin NAA or the ethylene biosynthesis precursor ACC, callus formation was induced in tobacco stems. In contrast, the ethylene biosynthesis inhibitor AVG partially inhibited NAA-induced callus formation, which demonstrated that the promoting effect of ethylene in grafting is likely due to its positive effect on callus formation in the scion-and-rootstock junction. Bars = 0.5 cm. The white-colored arrows point to the junctions of scions and rootstocks. The data are presented as the means from three independent biological replicates. The bars with different letters are significantly different at *P* < 0.05 (ANOVA, LSD). The asterisks (*) indicate a significant difference, as demonstrated by two-tailed Student’s t-test with pooled variance. The bars show standard deviations

Based on the transcriptome data, we also identified a series of ethylene-biosynthesis and ethylene-responsive genes that were differentially expressed during graft union formation (Table S1). For instance, the ethylene biosynthesis-related gene *ACS1* was upregulated 24 h after grafting in both the scion and rootstock of the WT/WT grafts compared with the level found at 0 h. However, the expression level of *ACS1* at 96 h after grafting was significantly reduced than that at 24 h after grafting (Fig. 4A). In the WT/*iaaM* grafts, *ACS1* expression also increased from 0 to 24 h after grafting and decreased from 24 to 96 h after grafting, and this finding was obtained in both the scion and rootstock tissues, but the increases in these grafts were more pronounced than those in the WT/WT grafts (Fig. 4A). The expression pattern of the ethylene-responsive gene *ERF5* was similar to that of *ACS1* in both the WT/WT and WT/*iaaM* grafts from 0 to 96 h after grafting (Fig. 4B). We also measured the 1-aminocyclopropane-l-carboxylic acid (ACC) levels in the scions of the grafts. In both the WT/WT and WT/*iaaM* grafts, the ACC levels in scion tissue also increased from 0 to 24 h and decreased from 24 to 96 h after grafting, and markedly greater changes were detected in the scions of the WT/*iaaM* grafts than in those of the WT/WT grafts (Fig. 4C). The dynamic changes in the ACC levels were similar to the changes detected in the expression patterns of *ACS1* and *ERF5*, which suggested that the ethylene levels might play a role in graft union formation. To verify the effects of ethylene on graft union formation, we applied the ethylene biosynthesis precursor ACC or the ethylene biosynthesis inhibitor 2-aminoethoxyvinylglycine (AVG) to the WT/WT graft junctions. Three weeks after grafting, scion growth was enhanced by ACC application but reduced by AVG application compared with the levels found with the control treatment (Fig. 4D–F). These results demonstrate that ethylene plays a positive role in grafting.

We then examined the involvement of ethylene in the action of auxin during grafting. Three weeks after grafting, the ethylene biosynthesis inhibitor AVG reduced scion growth in the WT/*iaaM* grafts (Fig. 4G). The dry biomass of scions was 4.3-fold higher in the WT/*iaaM* grafts than in the WT/WT grafts. In contrast, the AVG-treated WT/*iaaM* grafts exhibited 1.8-fold higher dry scion biomass than the WT/WT grafts (Fig. 4H). Therefore, the application of the ethylene biosynthesis inhibitor AVG delayed graft healing in the WT/WT grafts, but the delays were less pronounced in the WT/*iaaM* grafts, which suggested that ethylene inhibition partially inhibited the auxin-mediated improvement of graft union formation. We also explored the roles of auxin and ethylene in the callus formation process. After three weeks of treatment with NAA, a synthetic auxin, or ACC, an ethylene biosynthesis precursor, callus formation was induced in tobacco stems, whereas treatment with AVG, an ethylene biosynthesis inhibitor, partially inhibited NAA-induced callus formation, which demonstrated that the promoting effect of ethylene on grafting is likely due to its positive effect on callus formation in scion-and-rootstock junctions (Fig. 4I, J).

## Discussion

Grafting is widely used for both horticultural and scientific applications. In the current study, we examined the effects of using *SbUGT::iaaM* rootstock on WT scions during the grafting process in vitro. The overexpression of *iaaM* predominantly in the rootstock roots led to more rapid callus formation and faster graft union formation during the graft healing process compared with the results obtained with the WT rootstock. The use of *iaaM* rootstock led to high auxin levels in the grafted scions during graft healing, as revealed using *DR5::GUS* tobacco as the scion grafted onto WT or *iaaM* rootstock. Furthermore, the transcriptome analysis revealed that auxin-responsive genes were upregulated in the scions of the WT/*iaaM* grafts compared with those of the WT/WT grafts. Our findings also suggest that ethylene interacts with auxin to promote graft union formation. Our results thus shed light on the mechanism of graft union formation and could help increase the success rate of grafting.

A successful grafting process consists of three successive events: (i) cohesion of the scion and rootstock; (ii) proliferation of callus cells at the graft interface; and (iii) vascular redifferentiation across the graft interface^18,21,22^. The initial cohesion of the scion and rootstock is mediated by extracellular communication between the graft partners and involves activities such as the deposition and subsequent polymerization of cell wall materials^23^. In the current study, we observed thicker cell walls in the rootstock of the WT/*iaaM* grafts than in that of the WT/WT grafts, which might help strengthen the adhesion of the grafting partners^21,23,24^. Cell division or proliferation is needed for callus formation^25^. The formation of callus tissue at the graft junction is thought to represent a basic response to wounding during grafting, and a lack of callus formation results in graft failure^18,26,27^. Therefore, the rapid callus formation observed in the WT/*iaaM* grafts might be a critical factor in the fast graft healing process. For vascular development during *Arabidopsis* hypocotyl grafting, the initiation of phloem and xylem differentiation at different times results in time differences in connectivity. In addition, auxin promotes the formation of xylem and phloem cells^28,29^, which further facilitates the formation of graft unions.

The positive role of auxin in the partial incision healing process has been well demonstrated in the model plant *Arabidopsis* by exogenous application or decapitation^11^. Auxin is also involved in union formation in *Arabidopsis* hypocotyl grafting, which is not completely the same as wound healing^4,7^. Most studies have focused on auxin accumulation above the graft junction during grafting, whereas Melnyk et al^4^. found that auxin-responsive genes involved in vascular reconnection, such as *ALF4* and transport inhibitor response protein 1 (*TIR1*), are needed below the graft junction. Here, we showed that endogenous auxin in rootstock also plays an important role in grafting, as demonstrated with *SbUGT::iaaM* or *SAUR::iaaL* rootstock. Our results revealed that the auxin-mediated improvement of graft union formation is largely due to enhanced expression of genes important for callus and vascular cell development/reconnection. However, in *Arabidopsis* hypocotyl grafting, decreases in the endogenous auxin levels associated with *p35S::iaaL* (WT/*p35S::iaaL*) do not affect the phloem connection at 4 days after grafting^4^. The inconsistency of the results might be due to differences in organs or species.

Graft compatibility is needed for successful grafting^30^. Incompatibility between scions and rootstocks restricts the broad use of the grafting technique among different plant varieties^18^. In a compatible graft, the callus fills up the spaces between the scion-and-rootstock junction, which holds the two tissues together tightly, whereas a necrotic layer is always observed in incompatible grafts^31^. Auxin levels are correlated with graft compatibility^31,32^. Indeed, in a study of the homograft *Litchi chinensis* cv. ‘Jingganghongnuo’ (compatible graft) and the heterograft ‘Jing ganghongnuo’/‘zhuangyuanhong’ (incompatible graft), higher IAA levels were observed in compatible graft unions^31^. In the current study, we also found that increasing the endogenous auxin levels in the rootstock can improve the rate of successful grafting. Therefore, the overexpression of *iaaM* in rootstock has great potential for improving the rates of successful grafting with incompatible grafting partners.

Ethylene functions as a wounding response signal^14,33,34^. The ethylene-deficient *Arabidopsis* mutant *ein2* exhibits delayed tissue reunion compared with the WT in cut inflorescence stems^11^. Moreover, ethylene promotes callus formation in cultured cotton ovules by stimulating both cell division and cell expansion^35^. In the current study, we applied the exogenous ethylene biosynthesis precursor ACC and the ethylene biosynthesis inhibitor AVG to the WT/WT graft junctions. Our results demonstrated that ethylene plays a positive role in graft union formation. However, the ethylene-overproducing *Arabidopsis* mutant *ctr1-1* adversely affects the phloem connection in hypocotyl grafts^4^. A probable reason for this contradiction is the double-edged sword role of ethylene. Transgenic plants with elevated auxin levels also exhibited increased ethylene levels. An analysis of transcriptome data revealed that the expression of genes related to ethylene biosynthesis and responsive signaling pathways, such as 1-aminocyclopropane-1-carboxylate oxidase homolog 1 (Nitab4.5_0002916g0060.1), ethylene-responsive transcription factor (Nitab4.5_0007739g0010.1), and probable WRKY transcription factor 33 (Nitab4.5_0001226g0020.1), was markedly higher in the WT/*iaaM* grafts than in the WT/WT grafts. Additionally, AVG treatment partially delayed graft union formation in the WT/*iaaM* grafts, and callus formation induced by NAA treatment was inhibited by the application of AVG. These results suggest that ethylene is involved in the action of auxin during grafting and that the promoting effect of ethylene on grafting is likely due to its positive effect on callus formation in scion-and-rootstock junctions.

Numerous studies have suggested that communication between the scion and rootstock promotes graft healing. For instance, a signal exchange process between scion and rootstock cells has been observed 24 h after hypocotyl grafting in *Arabidopsis*^9^, but the detailed mechanism has not been well characterized. Because auxin can be transported short distances in a cell-to-cell manner through protoplasts, this phytohormone is thought to be transported across the graft junction prior to vascular reconnection^8,9^. Our transcriptome analysis identified a series of genes that are differentially expressed between the WT/*iaaM* and WT/WT scions prior to the formation of graft unions (24 h after grafting); although a number of genes, such as *Wound Induced Protein 1*^36^, *Lateral Organ Boundaries Domain 16* (*LBD 16*), *LBD17*, *LBD18*^37^, *Histone H4*^8^, *NAC096*, *NAC071*^7^, and *HCA2*^8^ have been identified, the exact mechanism responsible for graft union formation has not been illuminated. These genes function in multiple processes, including wounding responses, cell division, cell proliferation, vascular formation, and hormone responses. Among these processes, wounding responses are involved in graft union formation. *NRT3.1* (Nitab4.5_0003741g0010.1) was upregulated by 18.23-fold in the WT/*iaaM* compared with the WT/WT scions. This gene encodes a high-affinity nitrate transporter involved in jasmonic acid-independent wound signal transduction, which suggests its potential involvement in wounding responses during graft union formation. Nitrate can also function as a signaling molecule^38^. Although *NRT3.1* was upregulated in the scions of the WT/*iaaM* grafts compared with those of the WT/WT grafts, whether it participates directly or indirectly in auxin signaling pathways requires further study. By employing plants harboring the auxin reporter gene *DR5::GUS* as the scion and measuring the endogenous IAA levels, we demonstrated that increased auxin levels in the rootstock can lead to high auxin levels in the scions 24 h after grafting, i.e., before vascular reconnection, and this effect might be due to passive diffusion.

Although the *iaaM* rootstocks initially showed enhanced root biomass, more vigorous scion growth, and no lateral bud release compared with the WT rootstocks, the application of *SbUGT*::*iaaM* expression as a practical technology is not the best option due to an observed reduction in root growth^39^. Although we revealed the mechanism of graft union formation, we used *iaaM* plants to demonstrate that a high auxin content in the rootstock can result in a high auxin content in the scion regardless of vascular reconnection, and this effect is likely due to the upward transport or passive diffusion of auxin and accelerates callus proliferation and graft union formation. Our results suggest that auxin promotes graft union formation through interactions with ethylene, and these findings uncover great potential for the action of auxin during grafting.

**Fig. 5:**
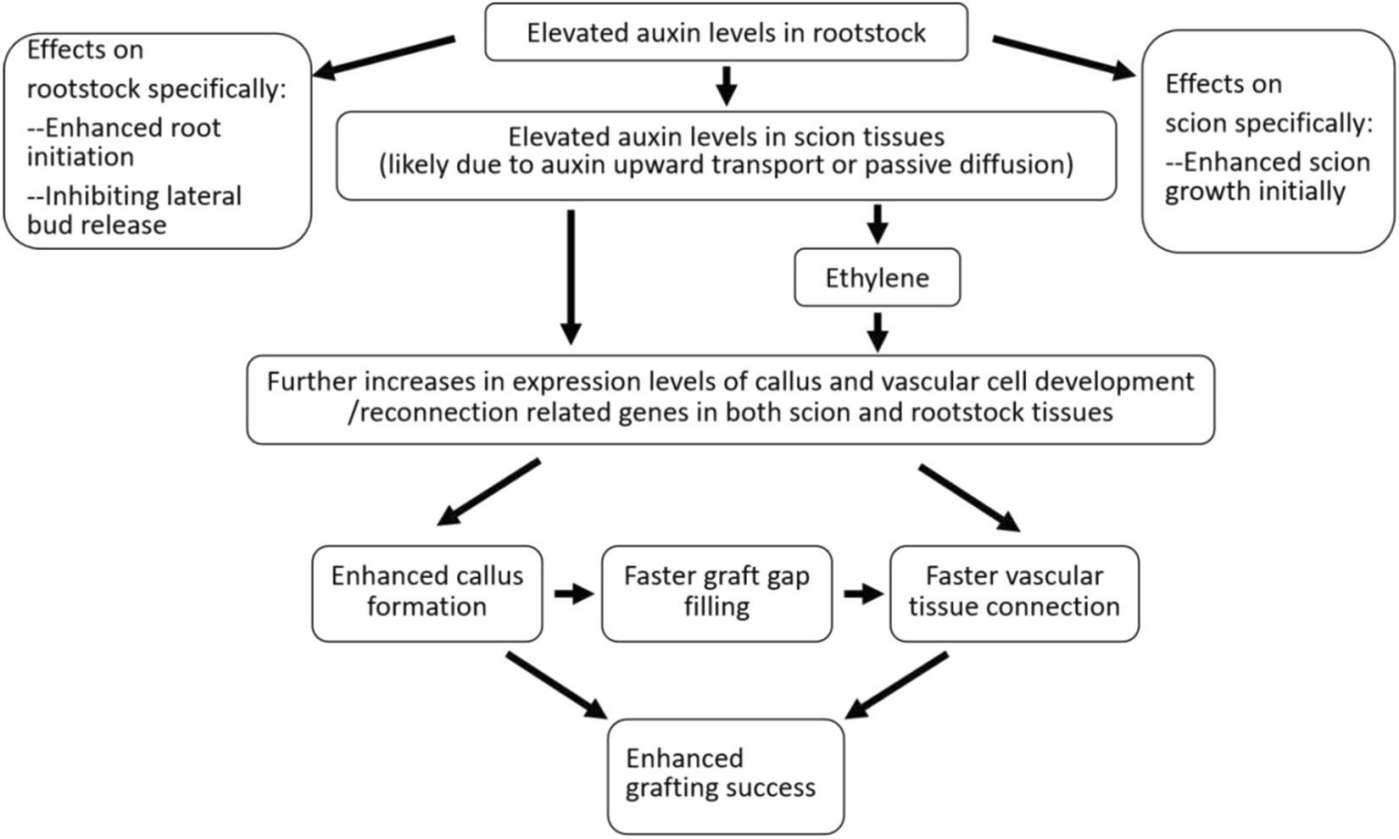
A working model for the actions of auxin-enriched rootstock during grafting. At the morphological and developmental levels, elevated auxin levels in rootstock tissues can exert a number of beneficial effects on rootstock and scion tissues. These effects on rootstock tissue include enhanced root initiation and inhibited lateral bud release, whereas the effects on scion tissue include more vigorous growth at early stages after grafting. At the biochemical and molecular levels, higher levels of auxin in rootstock tissue result in elevated auxin concentrations in scion tissues, which is likely due to the upward transport or passive diffusion of auxin from the rootstock to scion tissues. Higher auxin levels in rootstock tissues can enhance the expression of genes involved in callus formation and vascular tissue development/reconnection in both rootstock and scion tissues. Additionally, elevated auxin levels induce more ethylene production, and ethylene plays a positive role in callus formation and vascular tissue development/reconnection. Together, these effects lead to higher grafting success. The genes showing enhanced expression in rootstock tissue containing elevated auxin levels include *Wound induced protein 1* (*WIND1*)^36^, *Lateral Organ Boundaries Domain (LBD) 16*, *LBD17*, *LBD18*^37^, *Histone H4*^8^, cyclin genes^25^, such as *CYCA1-1*, CYCA2-2, *CYCB2-3*, *CYCB2-4* and *CYCD3-1*, the NAC domain-containing proteins *NAC096* and *NAC071*^7^, *high cambial activity 2* (*HCA2*)^8^, *expansin-A15*, and *expansin-like B1*

Based on the data presented in this manuscript, we propose a working model for the mechanism underlying the effects of auxin-enriched rootstock on grafting (Fig. 5). We propose that elevated auxin levels in rootstock tissues due to overexpression of a root-specific *iaaM* fusion gene can enhance grafting success through a series of physiological and molecular events in both rootstock and scion tissues. At the physiological and morphological levels, increased auxin levels enhance root initiation and inhibit lateral bud release in rootstocks, and these higher levels also promote more vigorous scion growth at early stages after grafting. At the biochemical level, elevated auxin concentrations in rootstock tissue also result in a high auxin content in scion tissues due to the upward transport or passive diffusion of auxin. Ethylene plays a positive role in callus formation and vascular tissue development/reconnection, and elevated auxin levels in rootstock tissue also induce more ethylene production in both rootstock and scion tissues. At the molecular level, we observed that the expression of a series of genes involved in callus formation, vascular tissue development, and vascular tissue connecting/graft union formation between rootstock and scion tissues, including *Wound induced protein 1* (*WIND1*), *Lateral Organ Boundaries Domain* (*LBD*) *16*, *LBD17*, *LBD18*, *Histone H4*, cyclin genes, such as *CYCA1-1*,CYCA2-2, *CYCB2-3*, *CYCB2-4* and *CYCD3-1*, the NAC domain-containing proteins *NAC096* and *NAC071*, *High Cambial Activity 2* (*HCA2*), *Expansin-A15*, and *Expansin-like B1*, are enhanced in both rootstock and scion tissues^7,8,25,36,37^ when *iaaM* transgenic rootstock is used.

## Materials and methods

### Plant materials and growth conditions

*SbUGT*::*iaaM* tobacco (*iaaM-39*, which was produced previously)^39^ and *SAUR::iaaL* tobacco (*iaaL* tobacco) were propagated on Murashige and Skoog medium (MS, Product ID M519) from PhytoTechnology Laboratories (USA). The plants were grown in a tissue culture room at 25 ± 2 °C with a 16-h photoperiod and a light intensity of 250 μmol quanta/m^2^/s. After two months of growth in MS medium, all *iaaM* tobacco and WT plants were used for grafting.

### Grafting experiments

WT, *iaaM*, and *iaaL* stems were used as rootstocks. WT apical shoots were used as scions. A scion with a wedge shape cut at the basal end was inserted into a rootstock with a longitudinal cut at the apical end (Fig. 3A). The WT/WT, WT/*iaaM*, and WT/*iaaL* grafts were placed into MS medium. Grafts with more than 1-cm increases in scion growth were considered to have been successfully grafted, and the grafting success rates for each type of graft were recorded. Root initiation requires continuous daily observation, and one week after grafting, the root development of each type of graft was photographed. Two weeks after grafting, scion growth and lateral bud release were observed and documented. We also created stem explants using a 2-cm longitudinal cut from the top of each stem section from the WT and *iaaM* plants and placed them on MS medium. One week after cutting, the spontaneous healing status was assessed and photographed.

### RNAseq sample and library preparation

Scion and rootstock tissues of the WT/WT or WT/*iaaM* grafts were harvested at 0, 24, and 96 h after grafting. Approximately 20 grafts from each graft combination at each time point were pooled to obtain one replicate, and data were collected from three biological replicates. RNA was extracted from the samples using RNeasy Plant Mini Kits, including the RNase-Free DNase set (Qiagen, Valencia, CA, USA). The degradation and contamination of RNA were monitored using 1% agarose gels, and the RNA purity was checked using a NanoPhotometer® spectrophotometer (Implen, CA, USA). The RNA integrity and quantitation were assessed using an RNA Nano 6000 Assay Kit and the Bioanalyzer 2100 system (Agilent Technologies, CA, USA). One microgram of RNA from each sample was used for library preparation. Sequencing libraries were generated using a NEBNext® Ultra™ RNA Library Prep Kit for Illumina® (NEB, USA) following the manufacturer’s recommendations. The library quality was assessed using the Agilent Bioanalyzer 2100 system.

### Bioinformatic analysis

The raw data (raw reads) were processed by removing adapter sequences. Clean reads were obtained by removing reads containing adapter and poly-N sequences and low-quality reads with low Q-values. The clean reads were mapped to the *Nicotiana tabacum* genome using TopHat v2.0.12. The gene expression levels were determined based on the fragments per kilobase of transcript sequence per million base pairs sequenced (FPKM) values. Differential expression analysis comparing two treatments was performed using the DEGSeq R package (1.20.0). A corrected *P*-value of 0.005 and a log_2_(fold change) value of 1 were used as thresholds to identify differentially expressed genes.

### Tobacco transformation and transgenic plant identification

The *DR5::GUS* construct^40^ was introduced into *Agrobacterium tumefaciens* strain EHA105, and the resulting bacteria were used to transform *Nicotiana tabacum* cv. Xanthi. Tobacco leaf disc transformation was performed as described previously^41^. Genomic DNA was extracted from the leaves of putative transgenic plants using a Plant Genomic DNA Extraction Kit (Macherey-Nagel, USA). The DNA was purified and used as a template for PCR^42^. The primer pair *GUS*-F (5′-TCTTCGACCTCAATGGCG-3′) and *GUS*-R (5′-ACGAATGACTTTTCCGAGG-3′) was used to amplify a 443-bp fragment from the *GUS* reporter gene within the T-DNA region of the Ti plasmid. PCR was performed using EmeraldAmp® GT PCR master mix (Clontech, USA). The reaction solution (20 µL) included 10 µL of 2× PCR mix, 0.5 µL of the forward primer, 0.5 µL of the reverse primer, 500 ng of the template DNA, and ddH_2_O. The PCR cycling conditions were as follows: 98 °C for 5 min; 35 cycles of 98 °C for 10 seconds, 60–65 °C for 30 seconds, and 72 °C for 1 minute/kb; and a final extension at 72 °C for 10 min.

### Histochemical GUS assays

Prior to grafting, *DR5*::*GUS* shoots were placed in MS medium for one day to induce the accumulation of endogenous auxin in the basal end. Approximately 0.5 cm of the shoot basal end was subsequently cut off, and the rest of the shoot was used as a scion for grafting onto WT or *iaaM* rootstock. Twenty-four hours after grafting, the scions were cut longitudinally in the middle of the stem and incubated in X-Gluc solution at 37 °C overnight for histochemical GUS staining. The X-Gluc solution consisted of 100 mM potassium phosphate buffer, pH 7.0, 10 mM Na_2_EDTA, 0.5 mM K_3_Fe(CN)_6_, 0.5 mM K_4_Fe(CN)_6_, 0.1% Triton X-100, and 1 g/L X-Gluc (5-bromo-4-chloro-3-indolyl-b-d-glucuronic acid). After staining, chlorophyll and other pigments were removed from the samples using successive concentrations of ethanol (100%, 75%, 50%, and 25%, the sample was incubated with each concentration for 12 h) prior to visual inspection and imaging. *SbUGT::GUS* and *35* *S::GUS* transgenic tobacco seeds^43^ were germinated in Petri dishes. After one week of growth, the seedlings were subjected to GUS staining as described above.

### Phytohormone analysis

The scions of the WT/WT or WT/*iaaM* grafts were harvested at 0, 24, and 96 h after grafting. Sample collection was performed as described for RNAseq analysis. The samples were weighed and frozen in liquid nitrogen. The IAA and ACC measurements were performed by HPLC-ESI-MSn (high-performance liquid chromatography coupled with electrospray ionization multi-tandem mass spectrometry) at Beijing Forestry University^44,45^. The mean hormone contents of the WT/WT and WT/*iaaM* grafts were compared using two-tailed Student’s t-test with pooled variance.

### Exogenous chemical treatments

Exogenous treatments were performed using the ethylene biosynthesis precursor 1-aminocyclopropane-1-carboxylic acid (ACC) (5 mM) and the ethylene biosynthesis inhibitor 2-aminoethoxyvinylglycine (AVG) (0.5 mM). Scions and rootstocks were treated with ACC or AVG prior to grafting, and treatment with water was included as the control. The cut scions and rootstocks were dipped into the treatment fluid for 3 seconds and then immediately grafted. Three weeks after grafting, the scion of each graft was harvested and dried at 105 °C for 30 min before weighing. The data are presented as the means from six replicates. Analysis of variance (ANOVA) was performed with IBM SPSS 19.0 (IBM Corp., Somers, NY, USA). Fisher’s protected least significant difference (LSD) test (*P* = 0.05) was used to calculate the differences between treatments.

### Tissue fixation and embedding

Tissue samples were incubated in a solution of 2.5% glutaraldehyde, 2.0% paraformaldehyde, 1.5 mM MgCl_2_, and 1.5 mM CaCl_2_ in 0.05 M PIPES, pH 6.9, for 3 h, transferred to fresh fixative and incubated at 4 °C for 3 h. The samples were subjected to three 20-minute rinses in 0.05 M PIPES (plus 1.5 mM MgCl_2_ and 1.5 mM CaCl_2_, pH 6.9), incubated overnight in fresh 0.05 M PIPES at 4 °C and rinsed for 40 min the next morning. Secondary fixation was conducted in 1% osmium tetroxide, 0.8% potassium ferricyanide, 1.5 mM MgCl_2_, and 1.5 mM CaCl_2_ in 0.05 M PIPES, pH 6.9, for 40 min at room temperature, and the samples were then incubated at 4 °C for 40 min. Prior to dehydration, the tissue was subjected to three 15-min rinses in cold Milli-Q distilled water (Milli-Q Academic System, Millipore). The tissue was dehydrated twice through a graded ethanol series (20 min for each concentration; 10%, 20%, 30%, 40%, 50%, 60%, 70%, 85%, and 100%) and cleared twice with propylene oxide for 10 min each time. Spurr’s resin (containing 3,4-epoxycyclohexane methyl 3ʹ,4ʹ-epoxycyclohexyl-carboxylate, DER 736 epoxy resin, nonenyl succinic anhydride, and 2-(dimethylamino)ethanol) was freshly prepared. The tissue was infiltrated in a 1:3 mixture of resin:propylene oxide for 1 h, a 1:1 mixture of resin:propylene oxide mixture for 3 h, and a 3:1 mixture of resin:propylene oxide overnight. The following day, the tissue was infiltrated in 100% Spurr’s resin for 7 h and incubated in fresh resin overnight. The next day, the tissue was infiltrated in 100% Spurr’s resin for 6 h. The samples were then placed in flat round molds (oriented longitudinally) and polymerized in an oven (Lab-Line Instruments, Inc.) under a vacuum at 60 °C for 48 h.

Semithin sections (~1 µm) were cut with a Histo 45° Diatome™ diamond knife on a Leica Ultracut UCT microtome and collected on drops of 10% acetone on Superfrost® Plus microscope slides (Fisher Scientific). The sections were dried by placing the slide drip-side down over a small open Petri dish (previously heated) for evaporation on a hotplate, stained at 70 °C with a solution of 1:1 methylene blue:azure blue II, and placed on a 30-8010 AB slide warmer (Buehler Ltd.) for 15 seconds at 90 °C. The sections were examined under an Olympus light microscope. All steps were conducted at room temperature in glass shell vials with plugs (Fisher Scientific) on a Pelco R2 rotary mixer (Ted Pella, Inc.) at setting 1 to facilitate penetration of the chemicals unless otherwise indicated.

### Callus induction and measurement

Tobacco internode stems (~2 mm) were placed on MS medium supplemented with 5 µM NAA, 10 µM AVG, or 10 µM ACC for callus induction. After three weeks, the callus was carefully removed with a scalpel and weighed. Eight to ten stems were grouped together to obtain one replicate. The data are presented as the means from three replicates. ANOVA was performed with IBM SPSS 19.0 software (IBM Corp., Somers, NY, USA). Fisher’s protected LSD test (*P* = 0.05) was used to calculate the differences between treatments.

## Supplementary information

Fig. S1 and S2

Table S1

## Data Availability

The RNA-seq datasets reported in our work have been submitted to the NCBI SRA database (BioProject ID PRJNA629440).
