# Supplementary material for: Molecular and physiological characterization of the effects of auxin-enriched rootstock on grafting
Source: Hortic Res. 2021 Apr 1;8:74. doi: 10.1038/s41438-021-00509-y (PMC8012700; doi:10.1038/s41438-021-00509-y)
Supplement: Supplementary file 1 — Fig. S1 and S2 [file 41438_2021_509_MOESM1_ESM.docx]

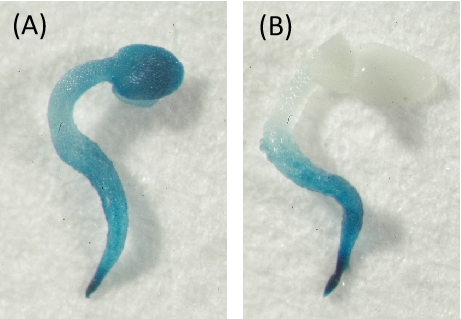


Fig. S1 The *SbUGT* promoter is predominantly expressed in roots. *SbUGT* promoter-driven *GUS* expression is specifically detected in roots (B) compared to constitutive *GUS* expression driven by the 35S promoter (A) in one-week-old seedlings.


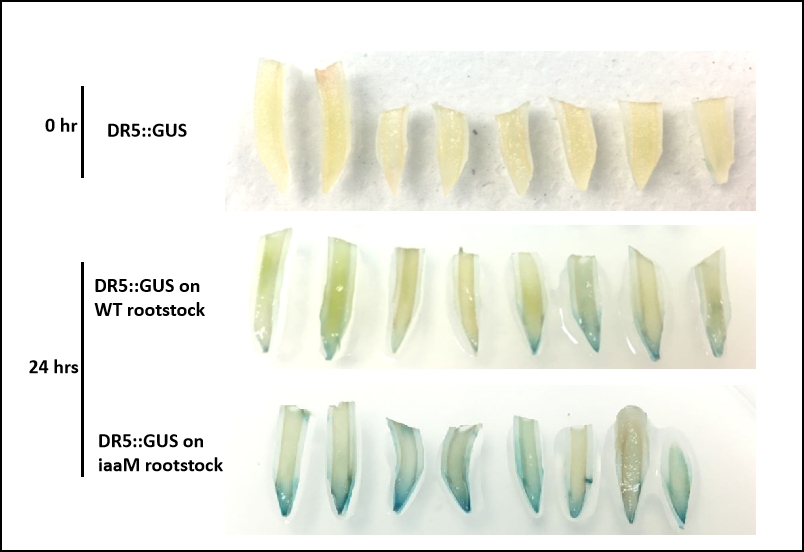


Fig. S2 *iaaM* rootstock enhances the GUS activity of *DR5::GUS* scions at the basal ends 24 hours after grafting. “0 hr” represents freshly cut shoots from *DR5::GUS* plants.
