# Supplementary material for: Molecular and physiological characterization of the effects of auxin-enriched rootstock on grafting
Source: Hortic Res. 2021 Apr 1;8:74. doi: 10.1038/s41438-021-00509-y (PMC8012700; doi:10.1038/s41438-021-00509-y)
Supplement: Supplementary file 2 — Table S1 [file 41438_2021_509_MOESM2_ESM.pdf]

WT scions were grafted on WT and iaaM rootstock, seperately. Scion and rootstock tissues were harvested at 0 hour, 24 hours and 96 hours, seperately.
Note: changes of gene expression level in WT scions on WT rootstock were presented in balck; while WT scions on iaaM rootstock in red.
Similarly, changes of gene expression level in WT rootstock were presented in black, while in iaaM rootstock in red

Tissues	Gene name	Gene ID	Changes in expression level in fold 0 hr		24 hrs		96 hrs		Gene function
**Callus formation related genes**									
WT sicons on WT or iaaM rootstocks	WIND1	Nitab4.5_0004997g0010.1	1.0	1.0	20.5	31.1	7.7	23.9	wound indcued; callus formation
	LBD18	Nitab4.5_0007537g0010.1	1.0	1.0	2.3	2.9	1.7	1.6	auxin and lateral root development
	LBD17	Nitab4.5_0004192g0020.1	1.0	1.0	3.3	28.2	14.2	80.7	auxin and lateral root development
	LBD16	Nitab4.5_0000086g0170.1	1.0	1.0	6.5	22.8	13.4	65.4	auxin and lateral root development
	Histone H4	Nitab4.5_0000270g0260.1	1.0	1.0	1.9	1.3	1.2	1.7	cell division
	Cyclin-A1-1	Nitab4.5_0006720g0060.1	1.0	1.0	1.2	1.2	1.9	2.4	cell division
	Cyclin-A2-2	Nitab4.5_0007182g0040.1	1.0	1.0	2.1	1.6	1.5	2.1	cell division
	Cyclin-B2-3	Nitab4.5_0010763g0020.1	1.0	1.0	1.2	1.1	1.7	2.4	cell division
	Cyclin-B2-4	Nitab4.5_0000588g0060.1	1.0	1.0	1.3	1.3	1.8	2.6	cell division
	Cyclin-D3-1	Nitab4.5_0006486g0030.1	1.0	1.0	0.9	2.0	2.0	3.4	cell division
	G2/mitotic-specific cyclin-1	Nitab4.5_0002967g0030.1	1.0	1.0	1.0	1.1	2.0	2.8	cell division
	G2/mitotic-specific cyclin-2	Nitab4.5_0008332g0040.1	1.0	1.0	2.6	2.4	3.2	4.4	cell division
	G2/mitotic-specific cyclin S13-6	Nitab4.5_0000428g0070.1	1.0	1.0	1.1	1.0	1.5	2.0	cell division
	Cyclin-P3-1	Nitab4.5_0006928g0020.1	1.0	1.0	3.0	2.3	4.7	1.9	cell division
WT or iaaM rootstocks with WT scions	WIND1	Nitab4.5_0004997g0010.1	1.0	4.2	7.9	12.9	16.2	22.5	wound indcued; callus formation
	LBD18	Nitab4.5_0007537g0010.1	1.0	1.9	0.6	1.6	0.3	1.1	auxin and lateral root development
	LBD17	Nitab4.5_0004192g0020.1	1.0	2.2	0.1	0.9	0	2.2	auxin and lateral root development
	LBD16	Nitab4.5_0000086g0170.1	1.0	1.2	0.1	0.1	0	2.0	auxin and lateral root development
	Histone H4	Nitab4.5_0000270g0260.1	1.0	2.3	3.2	9.3	2.5	6.3	cell division
	Cyclin-A1-1	Nitab4.5_0006720g0060.1	1.0	1.3	1.4	4.7	5.5	6.0	cell division
	Cyclin-A2-2	Nitab4.5_0007182g0040.1	1.0	1.2	2.0	8.2	5.6	7.0	cell division
	Cyclin-B2-3	Nitab4.5_0010763g0020.1	1.0	1.4	0.7	3.2	2.8	4.7	cell division
	Cyclin-B2-4	Nitab4.5_0000588g0060.1	1.0	1.1	0.8	3.2	2.7	4.1	cell division
	Cyclin-D3-1	Nitab4.5_0006486g0030.1	1.0	1.7	3.0	3.7	3.0	7.3	cell division
	G2/mitotic-specific cyclin-1	Nitab4.5_0002967g0030.1	1.0	0.5	0.6	3.3	2.6	3.5	cell division
	G2/mitotic-specific cyclin-2	Nitab4.5_0008332g0040.1	1.0	1.6	2.0	6.2	5.8	7.2	cell division
	G2/mitotic-specific cyclin S13-6	Nitab4.5_0000428g0070.1	1.0	1.3	1.1	3.8	3.0	3.7	cell division
	Cyclin-P3-1	Nitab4.5_0006928g0020.1	1.0	0.5	1.5	1.2	2.3	2.0	cell division
**Vascular development related genes**									
WT sicons on WT or iaaM rootstocks	Expansin-A15	Nitab4.5_0000141g0090.1	1.0	1.0	36.5	17.5	10.6	11.6	cell wall looseing
	Expansin-like B1	Nitab4.5_0000500g0210.1	1.0	1.0	123.1	315.0	42.1	219.9	cell wall looseing
	Expansin-like A1	Nitab4.5_0001274g0040.1	1.0	1.0	1.7	3.4	3.9	3.0	cell wall looseing
	Expansin-like B1	Nitab4.5_0003567g0050.1	1.0	1.0	124.4	253.9	57.2	176.9	cell wall looseing
	Expansin-A15	Nitab4.5_0005766g0020.1	1.0	1.0	32.2	15.4	9.9	3.0	cell wall looseing
	Cellulose synthase-like protein E1	Nitab4.5_0007779g0010.1	1.0	1.0	3.8	14.5	5.2	14.6	cell wall construction
	Caffeoyl-CoA O-methyltransferase 1	Nitab4.5_0002694g0030.1	1.0	1.0	2.2	3.5	1.1	3.8	cell wall reinforcement
	Caffeoyl-CoA O-methyltransferase 3	Nitab4.5_0002765g0010.1	1.0	1.0	1.0	2.0	0.8	2.0	cell wall reinforcement
	Laccase-4	Nitab4.5_0000119g0250.1	1.0	1.0	1.5	0.4	1.3	0.7	secondary xylem cell wall lignification.
	HCA2	Nitab4.5_0000003g0600.1	1.0	1.0	1.6	2.5	1.5	1.3	phloem formation
	NAC096	Nitab4.5_0003100g0030.1	1.0	1.0	23.9	71.6	25.9	21.8	vascularreconnection
	NAC071	Nitab4.5_0014378g0010.1	1.0	1.0	22.9	59.6	37.4	23.7	vascularreconnection
	VAN3-binding protein	Nitab4.5_0000678g0030.1	1.0	1.0	1.1	1.7	2.2	1.4	xylem formation
	TMO6	Nitab4.5_0000533g0150.1	1.0	1.0	2.3	5.6	7.1	4.5	provasculature formation
	Peroxidase 12	Nitab4.5_0000099g0090.1	1.0	1.0	3.8	13.5	2.3	22.5	second cell wall thickening
WT or iaaM rootstocks with WT scions	Expansin-A15	Nitab4.5_0000141g0090.1	1.0	64.0	126.6	5.7	30.2	15.0	cell wall looseing
	Expansin-like B1	Nitab4.5_0000500g0210.1	1.0	15.2	37.6	2.1	1.7	4.5	cell wall looseing
	Expansin-like A1	Nitab4.5_0001274g0040.1	1.0	1.1	1.0	1.7	1.4	1.5	cell wall looseing
	Expansin-like B1	Nitab4.5_0003567g0050.1	1.0	175.1	205.6	20.8	62.0	72.7	cell wall looseing
	Expansin-A15	Nitab4.5_0005766g0020.1	1.0	13.7	3.7	6.1	3.7	13.2	cell wall looseing
	Cellulose synthase-like protein E1	Nitab4.5_0007779g0010.1	1.0	4.5	1.4	3.5	2.5	5.4	cell wall construction
	Caffeoyl-CoA O-methyltransferase 1	Nitab4.5_0002694g0030.1	1.0	6.2	4.2	8.2	1.3	7.2	cell wall reinforcement
	Caffeoyl-CoA O-methyltransferase 3	Nitab4.5_0002765g0010.1	1.0	1.9	1.5	2.2	0.6	2.2	cell wall reinforcement
	Laccase-4	Nitab4.5_0000119g0250.1	1.0	0.1	0.4	0.1	0.0	0.1	secondary xylem cell wall lignification.
	HCA2	Nitab4.5_0000003g0600.1	1.0	0.8	3.3	8.2	2.2	7.1	phloem formation
	NAC096	Nitab4.5_0003100g0030.1	1.0	0.8	3.6	11.7	1.2	11.9	vascularreconnection
	NAC071	Nitab4.5_0014378g0010.1	1.0	1.0	8.6	13.6	2.6	8.3	vascularreconnection
	VAN3-binding protein	Nitab4.5_0000678g0030.1	1.0	0.5	0.9	1.2	2.0	1.9	xylem formation
	TMO6	Nitab4.5_0000533g0150.1	1.0	0.9	0.3	1.2	1.2	2.2	provasculature formation
	Peroxidase 12	Nitab4.5_0000099g0090.1	1.0	3.1	7.3	37.8	1.2	24.3	second cell wall thickening
**Ethylene biosynthesis and responsive genes**									
WT sicons on WT or iaaM rootstocks	ACCH	Nitab4.5_0002916g0060.1	1.0	1.0	2.1	15.7	24.3	72.1	ethylene synthesis
	NAC71	Nitab4.5_0014378g0010.1	1.0	1.0	22.9	59.6	37.4	23.7	ethylene signaling pathway
	WRKY transcription factor 33	Nitab4.5_0001226g0020.1	1.0	1.0	13.0	33.4	14.3	34.2	ethylene signaling pathway
	ERF92	Nitab4.5_0000777g0080.1	1.0	1.0	15.1	35.1	12.1	17.2	ethylene signaling pathway
	TIR1	Nitab4.5_0008145g0010.1	1.0	1.0	0.4	0.9	0.9	1.1	ethylene signaling
	Ethylene receptor 2	Nitab4.5_0004443g0040.1	1.0	1.0	1.1	2.5	2.3	3.6	ethylene receptor
	WRKY transcription factor 33	Nitab4.5_0000047g0140.1	1.0	1.0	23.8	50.1	27.3	65.9	ethylene signaling pathway
	ACS	Nitab4.5_0000915g0150.1	-	-	-	-	-	-	ethylene synthesis
	ACS	Nitab4.5_0000373g0240.1	1.0	1.0	3.0	7.9	5.9	4.5	ethylene synthesis
	ERF5	Nitab4.5_0007739g0010.1	1.0	1.0	1.9	4.5	0.9	3.3	ethylene signaling pathway
WT or iaaM rootstocks with WT scions	ACCH	Nitab4.5_0002916g0060.1	1.0	2.6	7.4	9.8	24.3	26.9	ethylene synthesis
	NAC71	Nitab4.5_0014378g0010.1	1.0	1.0	8.6	13.6	2.6	8.3	ethylene signaling pathway
	WRKY transcription factor 33	Nitab4.5_0001226g0020.1	1.0	1.3	7.3	11.3	5.4	11.7	ethylene signaling pathway
	ERF92	Nitab4.5_0000777g0080.1	1.0	1.9	10.9	8.7	6.8	5.6	ethylene signaling pathway
	TIR1	Nitab4.5_0008145g0010.1	1.0	0.7	0.2	0.2	0.2	0.5	ethylene signaling

Ethylene receptor 2	Nitab4.5_0004443g0040.1	1.0	0.5	1.1	1.5	1.5	1.9	ethylene receptor
WRKY transcription factor 33	Nitab4.5_0000047g0140.1	1.0	15.5	100.6	110.4	72.7	153.6	ethylene signaling pathway
ACS	Nitab4.5_0000915g0150.1	1.0	0.0	115.9	386.9	0.0	134.0	ethylene synthesis
ACS	Nitab4.5_0000373g0240.1	1.0	0.9	1.1	2.4	0.8	3.5	ethylene synthesis
ERF5	Nitab4.5_0007739g0010.1	1.0	1.1	2.6	5.1	2.1	3.8	ethylene signaling pathway
